# Ulinastatin Ameliorates Pulmonary Capillary Endothelial Permeability Induced by Sepsis Through Protection of Tight Junctions via Inhibition of TNF-α and Related Pathways

**DOI:** 10.3389/fphar.2018.00823

**Published:** 2018-08-13

**Authors:** Ming Fang, Wen-hong Zhong, Wen-liang Song, Yi-yu Deng, Duo-meng Yang, Bin Xiong, Hong-ke Zeng, Hua-dong Wang

**Affiliations:** ^1^Department of Pathophysiology, School of Medicine, Jinan University, Guangzhou, China; ^2^Department of Emergency and Critical Care Medicine, Guangdong General Hospital, Guangdong Academy of Medical Sciences, Guangzhou, China; ^3^Department of Emergency and Critical Care, Guangdong General Hospital’s Nanhai Hospital, Foshan, China; ^4^The Second School of Clinical Medicine, Southern Medical University, Guangzhou, China; ^5^Department of Burns, Guangdong General Hospital, Guangdong Academy of Medical Sciences, Guangzhou, China

**Keywords:** ulinastatin, sepsis, permeability, pulmonary capillary endothelial cells, tight junctions

## Abstract

**Background:** Increased permeability of pulmonary capillary is a common consequence of sepsis that leads to acute lung injury. In this connection, ulinastatin, a urinary trypsin inhibitor (UTI), is used clinically to mitigate pulmonary edema caused by sepsis. However, the underlying mechanism of UTI in alleviating sepsis-associated pulmonary edema remains to be fully elucidated. As tight junctions (TJs) between the pulmonary microvascular endothelial cells (PMVECs) play a pivotal role in the permeability of pulmonary capillary, this study investigated the effect of UTI on expression of junctional proteins in PMVECs during sepsis.

**Methods:** Male adult Sprague Dawley rats were subjected to cecal ligation and puncture (CLP) and divided into sham, CLP, and UTI+CLP groups. UTI was administered every 8 h for 3 days before CLP. At 48 h after surgery, Evans blue (EB) was administered to evaluate the pulmonary vascular leakage. Histological staining was used for evaluation of lung injury score. Using immunofluorescence staining and Western blot, the expression of junctional proteins (occludin, claudin-5, and ZO-1) in pulmonary endothelia was assessed. *In vitro*, PMVECs were divided into control, lipopolysaccharide (LPS), and UTI+LPS groups for examination of expression of junctional proteins and TNF-α as well as inhibitor of NF-κB (IκB), p38 mitogen-activated protein kinases (p38 MAPKs), c-Jun N-terminal kinases (JNKs), and extracellular signal-regulated kinases (ERKs) signaling pathways. Additionally, the expression of various junctional proteins was determined in PMVECs of control, LPS, and TNF-α receptor antagonist-LPS groups. PMVECs were also treated with TNF-α and TNF-α receptor antagonist and the expression of various junctional proteins was assessed.

**Results:** Compared with the CLP group, UTI markedly decreased EB leakage and lung injury score. The expression of occludin, claudin-5, and ZO-1 was decreased in both CLP rats and LPS-treated PMVECs, but it was reversed by UTI and TNF-α receptor antagonist. TNF-α expression was vigorously elevated in the lung of CLP rats and in LPS-challenged PMVECs, which were suppressed by UTI. In addition, TNF-α also reduced occludin, claudin-5, and ZO-1 expression in PMVECs, but these effects of TNF-α were antagonized by pretreatment with TNF-α receptor antagonist. Furthermore, UTI inhibited LPS-induced activation of NF-κB and mitogen-activated protein kinases (MAPKs) pathways in PMVECs.

**Conclusion:** UTI effectively protects TJs and helps to attenuate the permeability of pulmonary capillary endothelial cells during sepsis through inhibiting NF-κB and MAPKs signal pathways and TNF-α expression.

## Introduction

Mortality from sepsis is primarily attributed to injury and dysfunction of multiple organs (Gill et al., 2014). Among these, acute lung injury (ALI) and acute respiratory distress syndrome (ARDS) are the most frequent and fatal causes (Gill et al., 2015). The essential clinical features of ALI, including dyspnea, hypoxemia, bilateral infiltrates on chest radiograph, and consequent respiratory failure (Ranieri et al., 2012), are the results of the disruption of the alveolar-capillary barrier, leading to exudation of excessive fluid into the alveoli from pulmonary capillary which contributes to the fatal course of acute respiratory failure in clinical scenarios. The alveolar-capillary barrier or interface composed of alveolar epithelium and pulmonary capillary endothelium is the basis of oxygen-carbon dioxide exchange for normal respiration, and increase in pulmonary capillary endothelial cell permeability is deemed to play a crucial role in the development of sepsis-associated lung injury (Gonzales et al., 2015).

Pulmonary capillary endothelial cells or pulmonary microvascular endothelial cells (PMVECs) are tightly held with each other, thus forming a single and continuous layer as a barrier between blood and the pulmonary interstitium. Transcellular and paracellular routes are involved in the transport of macromolecules and fluids across the endothelial barrier (Birukov et al., 2013), whereas pathological vascular leakage usually takes place by paracellular routes (Park-Windhol and D’Amore, 2016). Bacterial endotoxins, cytokines, and other inflammatory mediators induce gaps between endothelial cells by disassembly of intercellular junctions (Lee and Slutsky, 2010), by altering the cellular cytoskeletal structure (e.g., microtubule disassembly) (Li et al., 2015), or by directly damaging the cell monolayer (e.g., PMVECs apoptosis) (Gill et al., 2015). The gaps resulting from microvascular leak and tissue edema are hallmark features of sepsis (Lee and Slutsky, 2010).

The inter-endothelial tight junctions (TJs) play a critical role in the paracellular permeability of the endothelial barrier (Komarova and Malik, 2010). TJs present in the epithelial, endothelial, and mesothelial cells are composed of more than 40 types of proteins that are either transmembrane proteins or cytoplasmic actin-binding proteins (Campbell et al., 2017). TJs connect neighboring cells with each other to create a barrier that regulates paracellular diffusion of ions and solutes and prevent pathogens from penetrating through the epithelial layers, thus serving as components of innate immunity (Soini, 2011). The primary constituents in the transmembrane group include the claudins, occludin, and junctional adhesion molecules (JAMs) (Campbell et al., 2017). Claudins are a family of tetraspan transmembrane proteins that form the structural basis for TJ permeability (Tsukita and Furuse, 2002; Gunzel and Fromm, 2012). Claudin-5 is most prominently expressed by the vascular endothelium and the pulmonary microcirculation is no exception (Kaarteenaho et al., 2010). Claudin-5 is likely to have a protective effect by increasing the barrier function of the endothelial part of the barrier (Chen H. et al., 2014). Occludin, an important regulator of TJ stability and function, biochemically interacts with claudins in TJ strands (Cording et al., 2013). Zonula occludens (ZO) is the primary cytoplasmic actin-binding proteins in TJs (Campbell et al., 2017). ZO-1 is a junctional adaptor protein that interacts with multiple junctional components, including the transmembrane proteins of nearly all claudins to link them to the actin cytoskeleton (Umeda et al., 2006) and control endothelial adherens junctions and endothelial barrier formation (Tornavaca et al., 2015). There is mounting evidence supporting the fact that the TJs of endothelial cells are reduced during inflammation, resulting in increased paracellular permeability as has been reported in the brain and umbilical veins (Rochfort et al., 2016; Ni et al., 2017).

Ulinastatin, urinary trypsin inhibitor (UTI), a potent multivalent Kunitz-type serine protease inhibitor derived from human urine, has been used clinically to treat acute pancreatitis and acute circulatory failure (Yoo et al., 2008). It has been reported that UTI suppresses various serine proteases such as trypsin, chymotrypsin, and plasmin *in vitro* and *in vivo* (Nishiyama et al., 1996) due to its properties of anti-inflammatory cytokines and anti-oxidative stress activities (Cao et al., 2012; Huang et al., 2013). Recently, UTI was demonstrated to exert protective roles against sepsis-induced multiorgan injuries (Zhou et al., 2008), such as septic ALI. There is strong evidence indicating that UTI inhibits oxidant-induced hyperpermeability in cultured human endothelial cells, in which apoptosis and JNK/c-Jun signaling pathway were involved (Li et al., 2014, 2016). Furthermore, post-treatment with UTI markedly decreased cytokines (TNF-α and IL-6) in the bronchoalveolar lavage fluid (BALF) and attenuated LPS-induced ALI in LPS-induced rat model (Luo et al., 2017). However, the protective effect of UTI on the tight junctional integrity of endothelia during sepsis-related lung injury and the underlying mechanisms have not been fully elucidated. In the present study, LPS-stimulated pulmonary endothelial cells and the cecal ligation and puncture (CLP) model in rats were adopted to investigate the effect of UTI treatment on the expression of pulmonary endothelial tight junctional proteins and the underlying mechanisms.

## Materials and Methods

### Ethics Statement on Use of Animals

In the handling and use of rats for CLP, ethical guidelines as stated in the National Institutes of Health Guide for the Care and Use of Laboratory Animals were followed. All experimental protocols and use of animals were approved by the approval authority at Jinan University (No. 20180225016), and all efforts were made to minimize the number of rats used and their suffering.

### Animals and Treatments

A total of 96 adult male Sprague Dawley rats (**Table 1**) weighing 250–300g were obtained from the Experimental Animal Center of Jinan University. They were kept in a 12 h light–dark cycle-controlled room, set at room temperature at 22–25°C and humidity (55 ± 15%), and fed with standard laboratory diet and water *ad libitum.*

**Table 1:** Number of rats used in various treatments.

	Sham	N.S+CLP(died in 48 h)	UTI+CLP(died in 48 h)
Lung pathology and WB	*N* = 5	*N* = 12(5)	*N* = 12(4)
EB	*N* = 5	*N* = 9(3)	*N* = 9(2)
Immunofluorescence	*N* = 5	*N* = 9(4)	*N* = 9(2)
ELISA	*N* = 5	*N* = 8(3)	*N* = 8(1)
Total	*N* = 20	*N* = 38(15)	*N* = 38(9)

The CLP surgical procedure in rats was performed as previously described (Rittirsch et al., 2009) to establish the model of pulmonary capillary leakage caused by sepsis. Briefly, following anesthesia with an intraperitoneal injection of 3% pentobarbital sodium (30 mg/kg), the abdominal cavity was opened via a midline laparotomy. The cecum was exposed and tightly ligated in the middle portion with a 3-0 silk suture and perforated 5 times with an 18-gauge needle (small amount of feces was exposed). After repositioning of the cecum, the abdomen was closed with a 2-0 silk suture. The rats were resuscitated by injecting pre-warmed normal saline (37°C; 5 ml/100g) subcutaneously. Beginning at 8 h post-CLP, the well-being and general conditions of rats (fur, posture, mobility, alertness, weight, and startle reflex) were assessed every 8 h. In the sham group, rats were subjected to similar surgical procedures, but the cecum was neither ligated nor perforated. To minimize variability between different experiments, the CLP procedure was always performed by the same investigator.

The rats were randomly divided into three groups: sham, CLP, and UTI+CLP. At 72 h before the CLP procedure, rats in the UTI+CLP group were given a tail intravenous injection of UTI (Techpool Biochemical Pharmaceutical Co., Ltd., Guangdong, China) (10000 U/kg dissolved in 2 ml 0.9% saline) every 8 h via an indwelling vein needle (24G, BD). The rats in the CLP group were given an equal volume of saline. In the sham group, rats were given neither normal saline (NS) nor UTI treatment. Vital signs (temperature, heart rate, and respiration rate) were recorded every 6 h. The clinical features of temperature and respiratory rate were recorded every 3 h till the rats were euthanized at 48 h after CLP.

### Assessment of Pulmonary Capillary Leakage

Protein extravasation, indicative of capillary permeability, was evaluated by measuring the extravasated Evans blue (EB). This method has previously been described by Sirois et al. (1990). In brief, at 48 h after CLP procedure, rats were anesthetized with an intraperitoneal injection of 3% pentobarbital sodium (30 mg/kg) followed by 2.5% EB (20 mg/kg, dissolved in 2 ml 0.9% saline) injection via the tail vein. Thirty minutes after the EB injection, the rats were euthanized. After this, the thorax was cut open and the lungs were perfused with 50 ml of phosphate buffered saline (PBS) (10 ml/min) via a cannula inserted in the pulmonary trunk to remove the excess of EB in pulmonary arteries. The right lung lobes for EB extraction were homogenized in formamide [4 ml/g (wet tissue)]. Following this, EB was extracted for 24 h at room temperature. The homogenates were centrifuged at 4000*g* for 30 min and supernatants assayed for EB concentrations. The dye absorbance value in the solution was then detected by a microplate reader (wave length, 620 nm), compared with a series of standard EB dilutions in formamide, and converted to mg of EB/mL of the solution. Based on this value, the amount of EB contained in the wet lung samples was then determined. All experiments were repeated at least in triplicate.

### Histological Examination and Lung Injury Score

At 48 h after CLP procedure, a total of 18 rats from all three groups were anesthetized with an intraperitoneal injection of 3% pentobarbital sodium (30 mg/kg) and the right middle lobe tissue was isolated and fixed by immersion in 4% paraformaldehyde, embedded in paraffin, and sectioned at 4 μm thickness. Following hematoxylin and eosin staining, pathological changes in lung tissues were examined under a light microscope. Lung injury score (LIS) was quantified by an investigator blinded to the treatment groups using recently published criteria (Matute-Bello et al., 2011). As shown in **Table 2**, the LIS was obtained by the sum of each of the six independent variables: neutrophils in the alveolar spaces and/or in the interstitial spaces, presence of hyaline membranes, proteinaceous debris filling the airspaces, alveolar septal thickening, and alveolar congestion. This sum was weighted according to the relevance ascribed to each feature by the American European Consensus Committee (Matute-Bello et al., 2011) and then was normalized to the number of fields evaluated and arbitrarily multiplied by 10 to obtain continuous values between zero and ten (both inclusive). Thus, the resulting LIS was derived from the following calculation: LIS = {[(20 × A) + (14 × B) + (6 × C) + (6 × D) + (2 × E) + (2 × F)]/(number of fields × 100)}× 10. All experiments were repeated at least in triplicate.

**Table 2:** Histological lung injury score.

Parameter^∗^	Score per field		
	0	1	2
A. Neutrophils in the alveolar space	None	1–5	>5
B. Neutrophils in the interstitial space	None	1–5	>5
C. Hyaline membranes	None	1	>1
D. Proteinaceous debris filling the airspaces	None	1	> 1
E. Alveolar septal thickening	<2×	2×–4×	>4×
F. Alveolar congestion	None	1–5	>5

*^∗^Lung injury scoring system [adapted from Matute-Bello] (Gonzales et al., 2015) Reprinted with permission of the American Thoracic Society. Copyright© 2016 American Thoracic Society.*

### Cell Culture

Primary rat PMVECs (BeNa Culture Collection, Suzhou, China, BNCC338210) were maintained in 75 cm^2^ culture flasks with completed medium composed of Dulbecco’s Modified Eagle Medium (DMEM, Gibco by Thermo Fisher Scientific, United States, C11995500BT) supplemented with 20% fetal bovine serum (FBS, Gibco by Life Technologies, United States, 10099-141), 1% antibiotic (Gibco by Life Technologies, United States, 15140-122), and heparin (90–100 U/ml) at 37°C in humidified 5% CO_2_/95% air. The medium was partially changed every 48 h for 10–14 days till the cells grew to confluence. For Western blotting and immunofluorescence labeling, cells were plated in six-well plates (3.5 × 10^5^ cells/well) and grown to confluence for experimental treatments.

### Cell Viability Assay

Viability of endothelial cells was assessed by Cell Counting kit-8 (CCK8; BestBio, Shanghai, China, BB-4202-2). To determine the cytotoxic effect of UTI, LPS, and TNF-α, PMVECs were plated into 96-well microplates (10^4^ cells/well) and cultured overnight. PMVECs were then treated with UTI (Ulinastatin for Injection, Techpool, Guangdong, China) (ranging from 2500 to 20000 U/ml), lipopolysaccharide (LPS, Sigma, United States, L-2880) (ranging from 0.5 to 32 μg/ml), rat tumor necrosis factor alpha (TNF-α, PeproTech, United States, 400-14) (ranging from 0.5 to 32 ng/ml) for 3, 6, 12, and 24 h in triplicates. After removal of medium, 90 μl of new basic medium and 10μl of CCK8 were added to each well and the plate was incubated for additional 3 h (final concentration 10%). The optical density (OD) was then read at 450 nm using a microplate reader. The assays were performed in triplicate.

### Cell Groups and Treatments

The PMVECs were randomly divided into three groups: control, LPS, and UTI+LPS. For detection of tight junctional proteins, PMVECs in the UTI+LPS group were pretreated with UTI (10000 U/ml) for 1 h and then incubated with basic medium + LPS (1 μg/ml) for 24 h, whereas the cells in LPS group were subjected to basic medium + LPS (1 μg/ml) for 24 h. In the control group, cells were incubated with basic medium for 24 h without any treatment. For TNF-α and signaling pathway determination, cells were pretreated with UTI at the same concentration for 1 h, but they were only incubated with LPS for 6 h and 30 min–1 h, respectively. The cells were then harvested for protein extraction or immunofluorescence microscopy (6-well plates). To further investigate the role of TNF-α in destruction of the tight junctional proteins, PMVECs were randomly divided into control, LPS, and TNF-α receptor antagonist (An) + LPS group. The cells in control and LPS group were prepared as mentioned above, whereas in An+LPS group, the cells were subjected to pretreatment with TNF-α receptor antagonist (0.2μM) (TNF-α receptor antagonist III, R-7050, Santa Cruz Biotechnology, Santa Cruz, CA, United States; sc-356159) for 30 min (Tornavaca et al., 2015) followed by basic medium + LPS (1 μg/ml) for 24 h.

To detect the tight junctional proteins given treatment of TNF-α and TNF-α receptor antagonist, PMVECs were randomly divided into control, TNF-α, and An+TNF-α group. PMVECs in TNF-α group were incubated with basic medium + TNF-α (10 ng/ml) for 18 h. In An+TNF-α group, the cells subjected to pretreatment with TNF-α receptor antagonist (0.2 μM) were incubated for 30 min followed by incubation with basic medium + TNF-α (10 ng/ml) for 18 h. In the control group, cells were incubated with basic medium for 18 h without any treatment. They were then harvested for protein extraction or immunofluorescence microscopy (6-well plates). All experiments were repeated at least in triplicate.

### Western Blotting

A total of 20 rats were used for Western blotting analysis. The left lung obtained from each group was frozen in liquid nitrogen and stored at −80°C. Tissue samples from various groups were homogenized with protein extraction reagent (Pierce; Thermo Fisher Scientific, Bloomingdale, IL, United States) containing protease inhibitors. For PMVECs of different groups, after the medium was discarded, the cell samples were rinsed more than twice with 1× PBS; the cells were lysed with the lysis buffer. The treated cells were mechanically scraped off with a rubber scraper and centrifuged at 13,000 rpm for 25 min and the supernatant was collected.

Protein concentrations of both tissues and PMVECs were determined by using Pierce TM BCA Protein Assay Kit (Thermo Fisher Scientific, United States, Prod # 23227). Samples of supernatant containing 40 μg protein of PMVECs were heated to 95°C for 5 min and then separated by sodium dodecyl sulfate-poly-acrylamide gel electrophoresis in 10% gels. Protein bands were electroblotted onto polyvinylidene difluoride (PVDF) membrane and blocked with 5% non-fat milk for 1 h at room temperature (for signaling pathway detection, proteins were blocked with 5% BSA for 1 h).

The membranes were incubated with primary rabbit polyclonal antibody occludin (Thermo Fisher Scientific, United States, #71-1500), claudin-5 (Thermo Fisher Scientific, United States, #35-2500), ZO-1 (Thermo Fisher Scientific, United States, #40-2200), and TNF-α (Abcam, United Kingdom, #ab6671), as well p38 MAPK Rabbit mAb (Cell Signaling Technology, United States, #8690S), Phospho-p38 MAPK Rabbit mAb (Cell Signaling Technology, United States, #4511S), p44/42 MAPK (Erk1/2) Rabbit mAb (Cell Signaling Technology, United States, #4370S), Phospho-p44/42 MAPK (Erk1/2) Rabbit mAb (Cell Signaling Technology, United States, #4695S), JNK Mouse mAb (R&D Systems, United States, #MB2076), Phospho-JNK Mouse mAb (Cell Signaling Technology, United States, 9255S), IκB Mouse mAB (Cell Signaling Technology, United States, #4814S), and Phospho-IκB Goat pAB (Santa Cruz Biotechnology, United States, #Sc-7977). Primary antibodies were diluted in 5% BSA overnight at 4°C before the membranes were incubated with the secondary antibodies, either with anti-rabbit IgG, HRP-linked antibody (Cell Signaling Technology, United States, 7074S) or anti-mouse IgG, HRP-linked antibody (Cell Signaling Technology, United States, 7076S). The immunoblots were detected by a SuperLumia ECL Kit (Abbkine, United States, K22020). The band intensity was quantified in Image J software (National Institutes of Health, NIH, United States). All experiments were repeated at least in triplicate.

### Double Immunofluorescence

A total of 17 rats from various experimental groups were used for double immunofluorescence labeling. Following deep anesthesia with 6% sodium pentobarbital, the rats were sacrificed by perfusion with 2% paraformaldehyde in 0.1 M phosphate buffer. The lung was removed, and paraffin was embedded. The inferior lobe of the right lung was sectioned at 7μm thickness on a microtome (Model: 2165; Leica, Bensheim, Germany). For blocking of non-specific binding proteins, tissue sections were incubated in 5% normal goat serum diluted in PBS for 1 h at room temperature (22 to 24°C). The sections were then incubated in a humidified chamber with the above mentioned primary antibodies diluted in PBS overnight at 4°C. Following washing in PBS, sections were incubated, with the respective fluorescent secondary antibodies: Cy3-conjugated secondary antibody and FITC-conjugated CD31 for 1 h at room temperature. After three rinses with PBS, the sections were mounted with a fluorescent mounting medium containing 4′,6-diamidino-2-phenylindole (DAPI) (Sigma, United States; Cat. No. F6057). Colocalization of various biomarkers was confirmed and images were captured using a confocal microscope (FluoView 1000, Olympus Company Pte. Ltd., Tokyo, Japan). The details of the antibodies used are given in **Table 3**.

**Table 3:** Antibodies used for Western blotting and immunostaining.

Antibody	Host	Source	Catalog number	Dilution for staining	Dilution for Western blot
CD31	Mouse monoclonal	Abcam, United Kingdom	ab64543	1:100	
Occludin	Rabbit polyclonal	Thermo Fisher Scientific, United States	71–1500	1:100	1:1000
Claudin5	Mouse monoclonal	Thermo Fisher Scientific, United States	35–2500	1:100	1:1000
ZO-1	Rabbit polyclonal	Thermo Fisher Scientific, United States	40–2200	1:100	1:1000
TNF-α	Rabbit polyclonal	Abcam, United Kingdom	Ab6671	1:100	1:1000
GAPDH	Mouse monoclonal	Biworld	MA001		1:3000
p-P38	Rabbit monoclonal	Cell Signaling Technology, United States	4511S		1:1000
P38	Rabbit monoclonal	Cell Signaling Technology, United States	8690S		1:1000
p-Erk	Rabbit monoclonal	Cell Signaling Technology, United States	4695S		1:1000
Erk	Rabbit monoclonal	Cell Signaling Technology, United States	4370S		1:1000
p-JNK	Mouse monoclonal	Cell Signaling Technology, United States	9255S		1:1000
JNK	Mouse monoclonal	R&D Systems, United States	MB2076		1:1000
p-IκB	Goat polyclonal	Santa Cruz Biotechnology, United States	Sc-7977		1:1000
IκB	Mouse monoclonal	Cell Signaling Technology, United States	4814S		1:1000
IgG-HRP	rabbit polyclonal	Cell Signaling Technology, United States	7074S		1:3000
IgG-HRP	Mouse monoclonal	Cell Signaling Technology, United States	7076S		1:3000
Secondary Antibody	Donkey polyclonal	Thermo Fisher Scientific, United States	A-31572	1:200	
Secondary Antibody	Donkey polyclonal	Thermo Fisher Scientific, United States	A-21202	1:200	

Pulmonary microvascular endothelial cells of various groups were seeded on coverslips in a 6-well plate (3.5 × 10^5^ cells/well) for 10–14 days till they grew to confluence. The medium was partially changed every 48 h. After different treatments, the PMVECs were fixed in 4% paraformaldehyde in 0.1 M PBS for 15 min at room temperature followed by blocking with 10% donkey serum for 1 h after rinsing with 1× PBS for 3 times at 5 min each. Subsequently, the coverslips with adherent cells were incubated in a humidified chamber with the respective primary antibodies as previously described overnight at 4°C, followed by the FITC/Cy3-conjugated secondary antibodies incubation for 1 h at room temperature. The coverslips were then mounted in DAPI containing the mounting medium after rinsing in 1× PBS for 3 times at 5 min each. Images were captured using a confocal microscope. All experiments were repeated at least in triplicate.

### ELISA

A total of 17 rats from various experimental groups were used for ELISA. TNF-α concentration of homogenates from the right lung in different groups was measured by TNF-α ELISA kit (R&D Systems, Inc, Minneapolis, MN, United States) according to the manufacturer’s protocol. The reaction plates were read within 30 minutes in an ELISA plate reader (Molecular Devices^®^, Eugene, OR, United States) at 450 nm.

### Statistical Analysis

Statistical analysis was performed by SPSS 16.0 statistical software. The data were expressed as mean ± SD. After homogeneity test of variances, one-way analysis of variance (ANOVA) followed by multiple comparison of Tukey test was used to determine the statistical significance of different groups. All experiments were conducted at least in triplicate from different cell or tissue samples. The difference was considered statistically significant when *p* < 0.05.

## Results

### UTI Prevented the Histopathological Changes in CLP-Induced Rat Lungs

**FIGURE 1:**
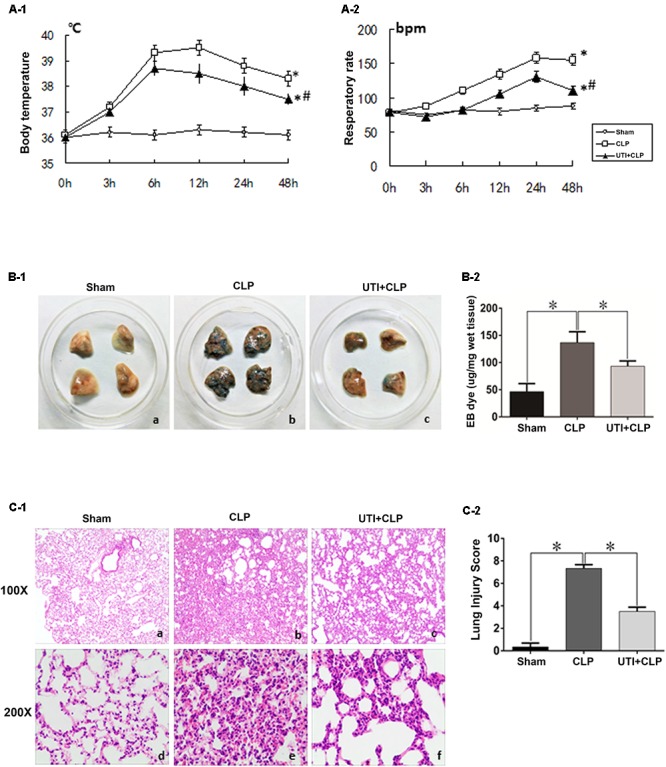
Clinical manifestations and histological assessment of the effects of urinary trypsin inhibitor (UTI) on cecal ligation and puncture (CLP)-induced sepsis rats. The temperature and respiratory rate of rats in both CLP and UTI+CLP groups showed a significantly higher level in comparison with the sham group at 48 h (^∗^**A-1** *p* = 0.000 and 0.0001, respectively; ^∗^**A-2** *p* = 0.000 and 0.000, respectively). Note that both readings were markedly decreased in UTI+CLP group compared with CLP group (^#^**A-1** *p* = 0.015 and ^#^**A-2** *p* = 0.001). The lung tissues from the sham group exhibited a normal color **(B-1a)** and gross features. There were detectable histopathological changes **(C-1a,d)**. In the CLP group, EB inundated the lung tissue **(B-1b)** (*p* = 0.000). In hematoxylin and eosin stained sections, severe pulmonary edema, hemorrhage in the stroma, alveolar collapse, and mass inflammatory cell infiltration were evident **(C-1b,e)** along with high lung injury score compared with the CLP group (LIC) **(C-2)** (*p* = 0.000). The extravasated EB in the UTI+CLP group was noticeably reduced compared with CLP group **(B-1c,B-2)** (*p* = 0.025). The UTI+CLP group showed a lesser destruction of lung structure **(C-1c,f)** compared with the CLP group. Furthermore, it showed a lower lung injury score than the CLP group **(C-2)** (*p* = 0.001). All values represent mean ± SD in triplicate; ^∗^ and # represents *p* < 0.05.

**FIGURE 2:**
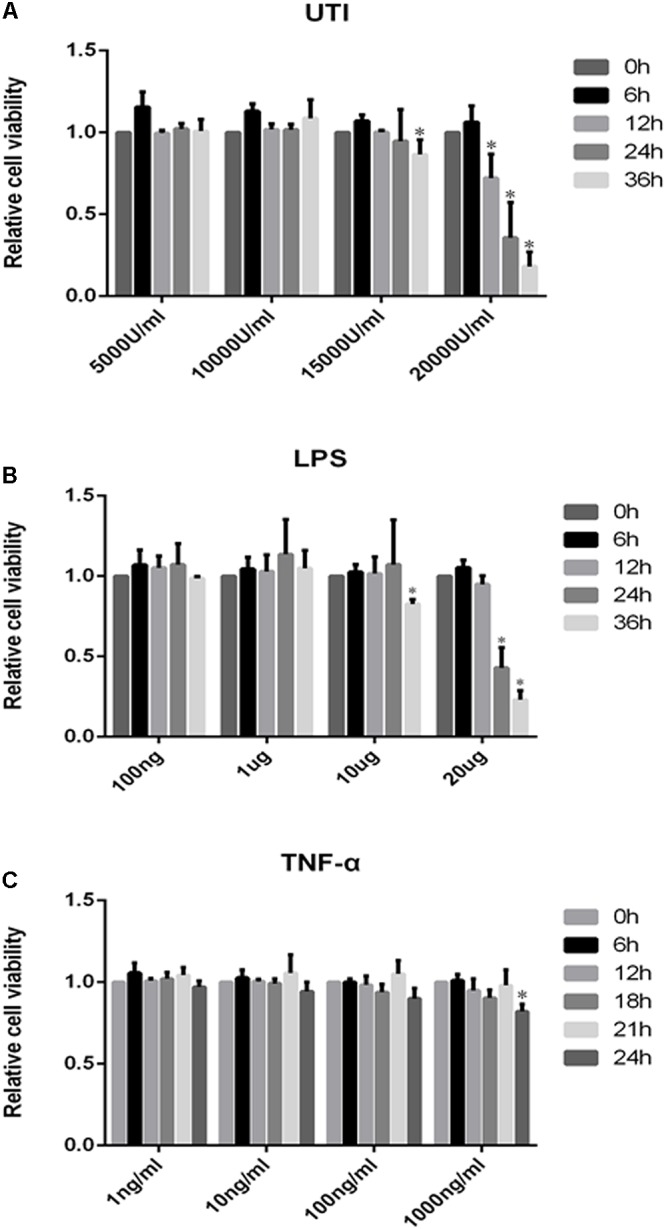
Effects of UTI, LPS, and TNF-α on viability of PMVECs. Showing cell viability of PMVECs treated with UTI **(A)**, LPS **(B)**, and TNF-α **(C)** at different doses and for different durations. In the range of 5000–10000μ/mL, UTI **(A)** did not affect the cells’ viability at 0–36 h; however, massive cell death occurred at 15000 U/mL of UTI at 36 h treatment (*p* = 0.006) and at 20000 U/mL at 12, 24, and 36 h (*p* = 0.002, 0.005, and 0.000, respectively). LPS **(B)** at concentration between 100 ng/mL and 1 μg/mL did not affect the cells’ viability at 0–36 h. When treated with LPS at 10 μg/mL for 36 h (*p* = 0.023) and 20 μg/mL for 24 and 36 h (*p* = 0.017 and 0.000), a higher incidence of cell death was detected in comparison with untreated controls. TNF-α **(C)** only at 1000 ng/mL could affect the cell viability at 24 h (*p* = 0.008). Considering the above, UTI at concentration of 10000 U/mL for 24 h, LPS 1 μg/mL for 24 h, and TNF-α 10 ng/mL for 18 h were adopted for subsequent *in vitro* studies. All values represent mean ± SD in triplicate; ^∗^represent significant differences, compared with corresponding control group, *p* < 0.05.

Of the 76 rats (see section “Materials and Methods”) that underwent CLP, 52 of them survived the surgical operation and were therefore considered as successful sepsis model for subsequent experimental analysis. Twenty four of them died within 48 h after operation and were excluded from the experimental analysis. As in clinical scenarios, the surviving CLP-induced sepsis rats presented with significantly raised temperature and respiratory rate compared with the sham group. UTI decreased the clinical indices in CLP-induced sepsis rats. In gross lung preparations, massive vascular exosmose of EB inundated the lung tissues after CLP. Following UTI treatment, the extravasated EB was markedly reduced (**Figures 1B-1,B-2**). Examination of hematoxylin and eosin-stained lung sections showed drastic histopathological changes caused by CLP as evident from the distinct thickening of alveolar septa, massive lymphocyte and neutrophils infiltrates in the interstitial spaces, hemorrhage in the stroma, pronounced alveolar collapse, and high LIS. However, in rats that were given UTI pretreatment, inflammatory infiltration in the lung tissue was reduced; additionally, there was diminution in destruction of lung structures and a significant decline in LIS when compared with the control rats that had not received UTI pretreatment (**Figures 1C-1,C-2**).

### Effects of UTI, LPS, and TNF-α on Viability of PMVECs

The cytotoxicity data were obtained by the MTS assay for the effect of UTI, LPS, and TNF-α on PMVECs. Cell viability remained relatively unchanged when PMVECs were exposed to UTI at 5000–10000 U/ml within 36 h (**Figure 2A**), to LPS at the concentration of 100–1000 ng/ml for 36 h (**Figure 2B**), and to TNF-α in the range of 1–1000 ng/mL for 24 h (**Figure 2C**), respectively. Only when cultured with UTI at 15000 U/ml for 36 h or at 20000 U/ml for more than 12 h, the viability of cells was significantly decreased (**Figure 2A**). At 10 μg/mL LPS for 36 h or 20μ g/mL LPS for 12 h, significant cell death was detected (**Figure 2B**). Considering the above, we have used UTI at concentration of 10000 U/mL for 24 h, LPS at 1 μg/mL for 24 h, and TNF-α at 10 ng/mL for 18 h for all subsequent *in vitro* analysis.

### UTI Enhanced Expression of Tight Junctional Proteins in Both CLP Rats and LPS-Treated PMVECs

**FIGURE 3:**
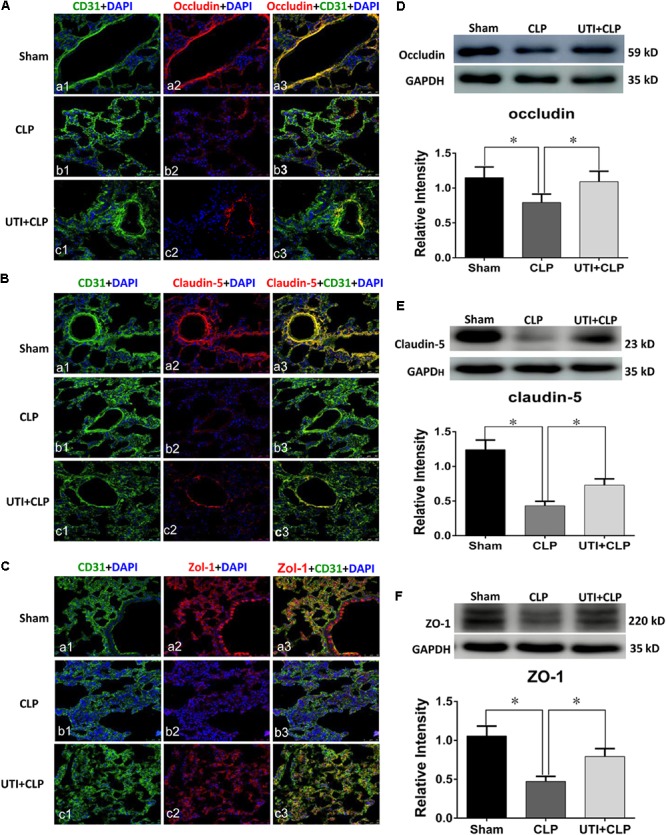
Urinary trypsin inhibitor enhanced expression of tight junctional proteins in CLP group. Confocal images showing occludin **(A)**, claudin-5 **(B)**, and ZO-1 **(C)** expression in rat lungs. Occludin, claudin-5, and ZO-1 expression (red) was intensely labeled in CD31 positive PMVECs (green) of sham group lung tissues **(Aa1–3,Ba1–3,Ca1–3)** but was hardly detected in CLP group **(Ab1–3,Bb1–3,Cb1–3)**. The expression was markedly increased in CLP rats treated with UTI **(Ac1–3,Bc1–3,Cc1–3)**. Western blot showing expression level of occludin **(D)**, claudin-5 **(E)**, and ZO-1 **(F)** that was significantly decreased in CLP when compared with the control (*p* = 0.001, 0.000, and 0.001, respectively); it was significantly augmented when pretreated with UTI (*p* = 0.018, 0.021, and 0.026, respectively). Scale bars: 50 μm, DAPI-blue. All values represent mean ± SD in triplicate; ^∗^represent significant differences, *p* < 0.05.

**FIGURE 4:**
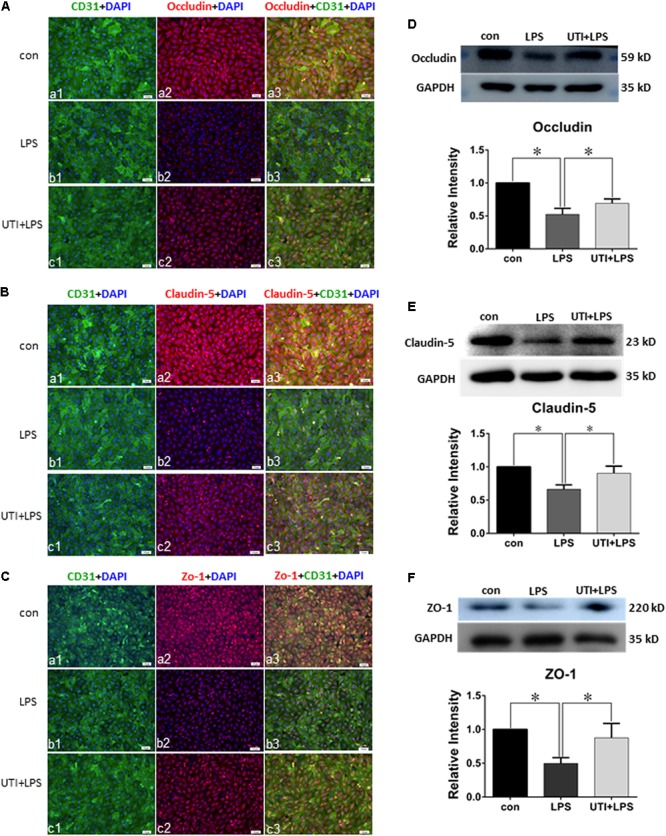
Urinary trypsin inhibitor enhanced expression of tight junctional proteins in LPS-treated PMVECs. Confocal images showing expression of tight junctional proteins in PMVECs. Increased occludin **(Aa1–3)**, claudin-5 **(Ba1–3)**, and ZO-1 **(Ca1–3)** expression (red) was detected in CD31 positive PMVECs (green) of control group. It was hardly detected in LPS group **(Ab1–3,Bb1–3,Cb1–3)**. The expression was markedly enhanced in LPS group that was agiven UTI pretreatment **(Ac1–3,Bc1–3,Cc1–3)**. Western blot shows expression levels of occludin **(D)**, claudin-5 **(E)**, and ZO-1 **(F)**. Note that the expression of all three proteins was significantly decreased in LPS when compared with control (*p* = 0.000, 0.001, and 0.001, respectively); it was significantly reversed to levels comparable to that of the control when pretreated with UTI (*p* = 0.000, 0.004, and 0.006, respectively). Scale bars: 50 μm, DAPI-blue. All values represent mean ± SD in triplicate; ^∗^represent marked differences compared with corresponding control.

Expression of various junctional proteins (occludin, claudin-5, and ZO-1) was assessed in CLP rat lungs (**Figure 3**) and PMVECs (**Figure 4**). By Western blotting and immunofluorescence labeling, intense occludin expression was detected in the lung tissue of sham rats. In rats subjected to CLP, occludin expression was hardly detected (**Figure 3A**). On the other hand, in CLP rats that were given UTI treatment, occludin expression was restored and enhanced by almost twofold (**Figure 3D**). Expression changes of occludin in PMVECs followed that in the rat lungs. Thus, in comparison with the matching controls, occludin immunofluorescence and its protein expression level as determined by Western blot was diminished or decreased in LPS-treated cells. The decline was significantly reversed by a regain of 40–50% increase in UTI pretreatment cells (**Figsures 4A,D**).

In parallel to occludin, expression of claudin-5 (**Figures 3B,E**) and ZO-1 (**Figures 3C,F**) was noticeably decreased in the lungs of CLP rats. A striking feature after UTI pretreatment was the pronounced increase in claudin-5 (**Figure 3B**) and ZO-1 (**Figure 3C**) immunofluorescence. Additionally, claudin-5 and ZO-1 protein levels rose by 50–100% as shown by Western blot when compared with the respective CLP group in the absence of UTI (**Figures 3E,F**). In PMVECs of UTI+LPS group, immunofluorescence intensity of claudin-5 (**Figure 4B**) and ZO-1 (**Figure 4C**) was evidently increased when compared with the respective LPS group; increase in claudin-5 protein level by Western blot was about 50% when compared with the LPS group (**Figure 4E**); ZO-1 intensity was also substantially increased to approximately 100% when compared with the LPS group (**Figure 4F**).

### TNF-α Expression Was Enhanced by Sepsis and Inhibited by UTI

**FIGURE 5:**
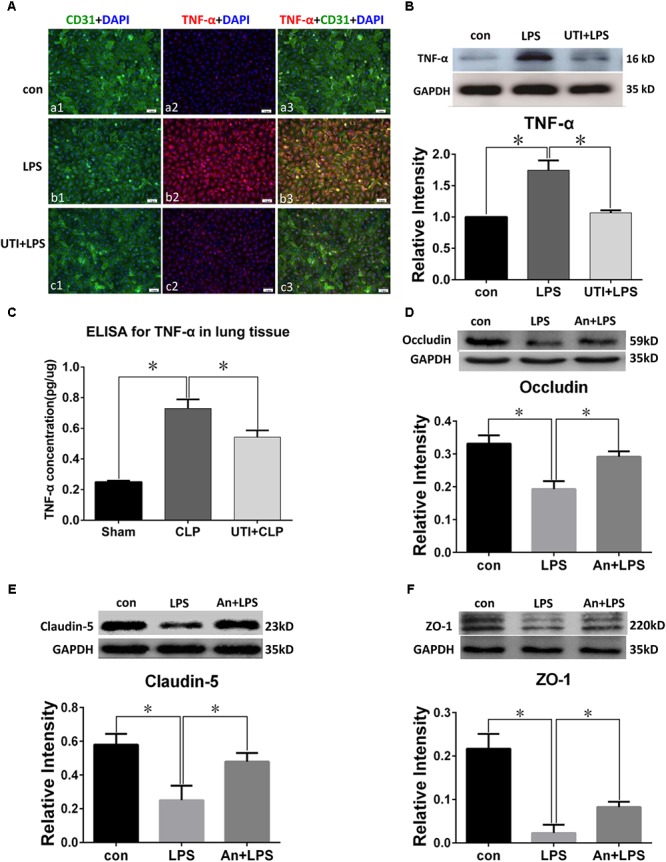
TNF-α expression was decreased by UTI in LPS-treated PMVECs and CLP lung tissue. Confocal images show TNF-α **(A)** expression (red) was markedly enhanced in PMVECs (green) of LPS group **(Ab1–3)** compared with control **(Aa1–3)** but obviously reduced when UTI pretreatment was given **(Ac1–3)**. Western blot showing expression level of TNF-α **(B)** was significantly increased by LPS (*p* = 0.002) and reversed when pretreated with UTI (*p* = 0.003). ELISA analysis showing expression level of TNF-α **(C)** was significantly increased in CLP group compared with sham (*p* = 0.003); it was significantly decreased when pretreated with UTI (*p* = 0.007). Western blot showing expression level of occludin **(D)**, claudin-5 **(E)**, and ZO-1 **(F)** that was significantly decreased in LPS group when compared with the control (*p* = 0.006, 0.001, and 0.001, respectively); it was significantly augmented when pretreated with TNF-α receptor antagonist (An+LPS) (*p* = 0.024, 0.017, and 0.021, respectively). Scale bars: 50 μm, DAPI-blue. All values represent mean ± SD in triplicate; ^∗^ represent significant differences compared with corresponding control.

Lipopolysaccharide treatment elicited a vigorous TNF-α expression in PMVECs as revealed by immunofluorescence (**Figure 5A**). Upregulated TNF-α expression was confirmed by Western blot which showed an increase by more than twofold in comparison with the control (**Figure 5B**). Interestingly, TNF-α expression was suppressed by as much as 50% when PMVECs were given UTI pretreatment (**Figure 5B**).

Because TNF-α concentration in the rat lung homogenates was too low to be detected, we had used the TNF-α ELISA kit for more accurate analysis. TNF-α (**Figure 5C**) concentration was increased by more than double in sepsis-induced group compared with the sham group; in UTI pretreatment group, it was suppressed by about 40%.

### TNF-α Decreased the Expression of Tight Junctional Proteins in PMVECs

Western blot in PMVECs showed the expression level of occludin (**Figure 5D**), claudin-5 (**Figure 5E**), and ZO-1 (**Figure 5F**) was significantly decreased in LPS group by 40–80% when compared with the control; it was significantly augmented when pretreated with TNF-α receptor antagonist (An+LPS group).

**FIGURE 6:**
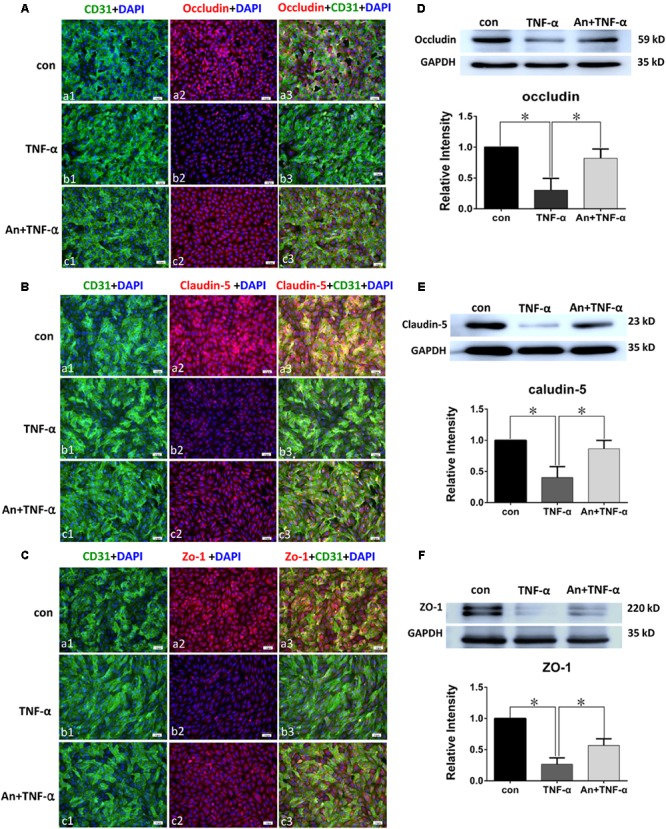
TNF-α receptor antagonist enhanced expression of tight junctional proteins in TNF-α treated PMVECs. Confocal images showing occludin **(A)**, claudin-5 **(B)**, and ZO-1 **(C)** expression in PMVECs. Increased occludin, claudin-5, and ZO-1 expression (red) was detected in CD31 positive PMVECs (green) of control group **(Aa1–3,Ba1–3,Ca1–3)**. It was hardly detected in TNF-α group **(Ab1–3,Bb1–3,Cb1–3)**. The expression was markedly increased when TNF-α receptor antagonist was given **(Ac1–3,Bc1–3,Cc1–3)**. Western blot shows expression level of occludin **(D)**, claudin-5 **(E)**, and ZO-1 **(F)** were significantly decreased in TNF-α when compared with control (*p* = 0.001, 0.000, and 0.001, respectively); it was significantly reversed to levels comparable to that of the control when pretreated with TNF-α receptor antagonist (*p* = 0.004, 0.003, and 0.002, respectively). Scale bars: 50 μm, DAPI-blue. All values represent mean ± SD in triplicate; ^∗^represent marked differences compared with corresponding control.

Immunofluorescence intensity of occludin in TNF-α treatment group was significantly decreased in comparison with PMVECs control (**Figures 6Aa1–3,b1–3**), whereas it was markedly augmented by TNF-α receptor antagonist in An+TNF-α group (**Figure 6Ac1–3**). Similar changes were determined for Claudin-5 (**Figure 6B**) and ZO-1 (**Figure 6C**). In PMVECs as assayed by Western blot, the protein expression level of tight junctional proteins including occludin (**Figure 6D**), claudin-5 (**Figure 6E**), and ZO-1 (**Figure 6F**) was significantly decreased by 50–80% following TNF-α treatment compared with the control group. Very strikingly, it was markedly elevated to a level comparable to that of the control cells when pretreated with TNF-α receptor antagonist (**Figures 6D–F**).

### Potential Signaling Pathways Involved in UTI Pretreatment

**FIGURE 7:**
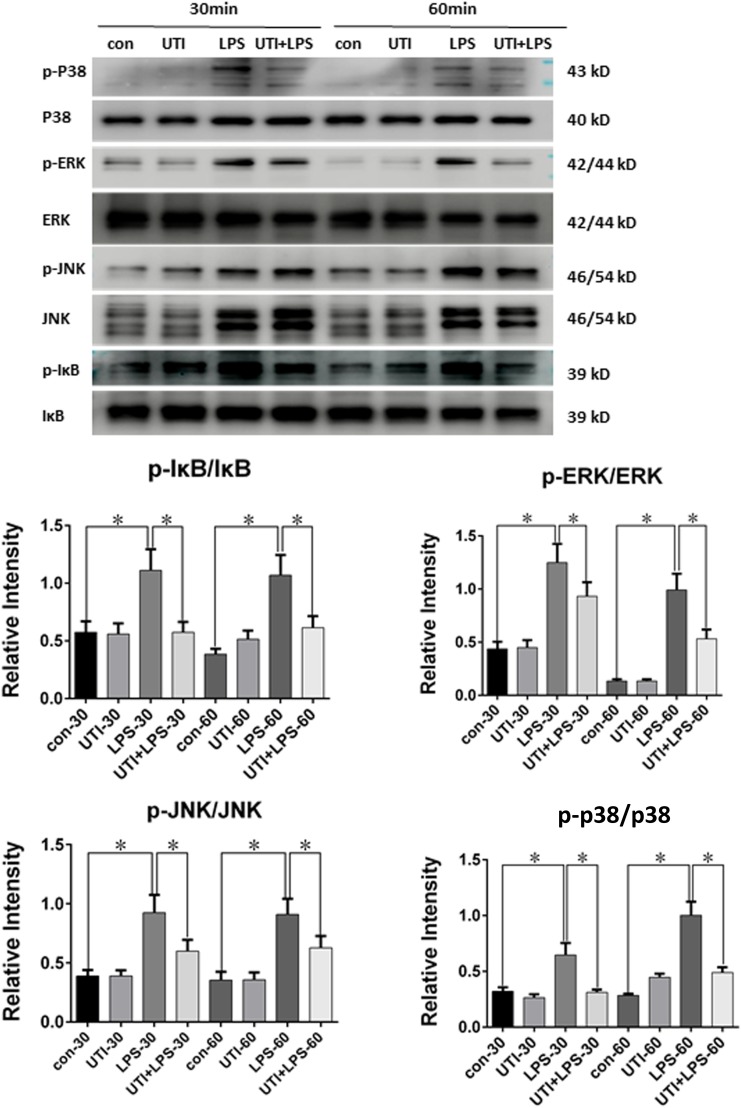
Protein expression of p-P38/P38 MAPK, p-IκB/i-κb, p-JNK/JNK, and p-ERK/ERK was decreased in LPS-treated PMVECs following UTI pretreatment. p-P38/P38 MAPK, p-IκB/i-κb, p-JNK/JNK, and p-ERK/ERK expression in PMVECs was increased significantly at 30 min (*p* = 0.001, 0,002, 0.000, and 0.013, respectively) and 60 min (*p* = 0.001, 0.000, 0.001, and 0.0012, respectively) following treatment with LPS when compared with the control group; it was markedly decreased when given UTI (*p* = 0.015, 0.012, 0.033, and 0.019, respectively at 30 min; *p* = 0.022, 0.009, 0.018, and 0.015, respectively at 60 min). Significant differences in protein levels are expressed as ^∗^*p* < 0.05. The values represent the mean ± SD in triplicate.

Compared with the control group, the ratio of p-P38/P38, p-IκB/i-κb, p-JNK/JNK, and p-ERK/ERK in PMVECs was increased significantly at 30 min and 60 min following LPS treatment; it was markedly depressed when given UTI treatment (**Figure 7**).

## Discussion

Self-infection experimental models in which animals are infected by endogenous pathogens are widely accepted because they share many of the clinical manifestations such as hemodynamic, metabolic, and immunological features of human sepsis. In the present study, we have used the sepsis model of CLP in rats for subsequent experimentation *in vivo*. Unlike the other commonly used sepsis model, such as exogenous administration of lipopolysaccharide, zymosan or viable pathogen (bacteria) by intraperitoneal injection, or intravascular injection(Buras et al., 2005), CLP model is a typical self-infection model. It characterizes the onset of sepsis secondary to local abdominal infection originated from the leaked cecum induced by surgery of ligation and puncture on the cecum. CLP model closely simulates the human diseases of ruptured appendicitis or perforated diverticulitis, which are the common causes of sepsis in clinic; more importantly, it triggers immediate systemic activation of the inflammatory response, causes the typical symptoms of sepsis, such as high temperature, tachycardia, and tachypnea. As similar hemodynamic, metabolic, and immunological features of human sepsis are observed in CLP model, it is the experimental animal model of choice for sepsis research (Wichterman et al., 1980; Rittirsch et al., 2009).

In this study, the middle portion of the appendix was perforated five times with an 18-gauge needle in each individual to ensure the reproducibility outcomes. The mortality rate of CLP rats in 48 h was about 32%, indicating severe sepsis as described in a previous study (Dejager et al., 2011). As shown in **Figure 1**, CLP rats presented with significant increase in temperature and respiratory rate compared with the sham group, which is in accordance with the clinical scenarios. Therefore, it can be confidently stated that CLP rat model used in this study is valid and reliable as it closely mimics sepsis in clinic.

Previous studies have reported that UTI treatment improved the outcome in clinical scenarios and animal models of sepsis, through inhibiting the inflammatory response, relieving the sepsis-induced pulmonary capillary leakage, and lung edema (Wang et al., 2013; Chen W. et al., 2014). Of note, the present results have shown that UTI administration effectively ameliorates the clinical symptoms of CLP rats (**Figure 1A**). Furthermore, UTI administration decreased the exosmose of Evans blue, which otherwise would have inundated the lung tissues in CLP-induced rats as revealed by gross examination and pathological LIS based on light microscopy (**Figures 1B,C**). It is well known that the vascular endothelium possesses some of the properties of inflammatory cells and could be activated by cytokines or endotoxin caused by infection, injury, or cancer (Armstrong et al., 2013). One of the characteristic features of activation of endothelium is the loss of vascular integrity followed by exposure of subendothelium and the efflux of fluids from the intravascular space (Blann, 2000). This would lead to significant and sustained increase in vascular permeability, a characteristic feature of acute inflammatory processes and constitute the essential basis of oxygenation dysfunction in ALI or ARDS secondary to sepsis (Wang et al., 2011). The definite target and additional mechanisms whereby UTI can exert its protective effects against septic lung injury has remained dubious. Nonetheless, the tight junctional proteins associated with the pulmonary microvascular endothelia have been considered to be the likely target. Considering the above and the protective effects exerted by UTI on sepsis-induced lung injury, we postulate that the therapeutic target of UTI may be associated with the endothelial TJs. The present results have shown (**Figure 3**) that the expression of tight junctional proteins such as occludin, claudin-5, and ZO-1 was decreased in the lung tissues of CLP-induced sepsis rats when compared with the sham group. Remarkably, UTI pretreatment reversed such changes; in other words, the expression of the above mentioned tight junctional proteins was restored to a varying extent. To confirm this, we have extended the study to rat primary cultured endothelia cells challenged with LPS. Results *in vitro* (**Figure 4**) were consistent with those *in vivo*. It is, therefore, suggested that during lung infection, activated endothelial cells undergo morphological changes along with biochemical changes of TJs, which conceivably would contribute to endothelia barrier disruption and inflammatory leakage. In the lung, endothelial cells serve as a semi-permeable barrier between the vascular contents and the pulmonary airspaces and play a critical role in regulating the tissue fluid homeostasis and the inflammatory response. There is supporting evidence that TJs play a critical role in the integrity alveolar-endothelia barrier (Dudek and Garcia, 1985). Decrease in TJ molecules in the alveolar epithelia and endothelia such as occludin, ZO-1, claudins, and VE-cadherin following inflammation typically leads to increased lung vascular permeability (Peter et al., 2017). It is noteworthy that moderate claudin-5 expression was detected in the endothelial cells. Reduction in claudin-5 or other adhesion molecules could lead to increased passage of fluid and cells into the alveolar space resulting in poor gas exchange (Armstrong et al., 2012). It is relevant to note that in the studies of the blood–brain barrier, it has been shown that UTI protected the brain against cerebral ischemia/reperfusion injury through reversing the loss of ZO-1 and occludin in the brain endothelia in mice with middle cerebral artery occlusion (Li X. et al., 2017). Separately, UTI was reported to enhance the expression levels of claudin-5 and ZO-1 in the brain capillaries and alleviate the inflammation in the hippocampus of aged rats following partial hepatectomy (Ma et al., 2016). Since the changes of tight junctional proteins were in synchrony with EB exosmose and pathological LIS observed *in vivo*, it can be confidently concluded that UTI can ameliorate the permeability of pulmonary capillary endothelial cells challenged with sepsis through protection of TJs.

Considering the above mentioned, a pertinent question would be the underlying mechanism by which UTI can exert its protective effect on TJs in the pulmonary endothelia following sepsis. To this end, we have shown that TNF-α expression in PMVECs was drastically decreased by UTI treatment in LPS-activated PMVECs, and similar changes were detected in the lung tissue of CLP rat (**Figure 5**). TNF-α, a key early signaling molecule in the inflammatory cascade, is a cytokine released not only by the activated immune cells such as monocytes and macrophages but also by the activated endothelial cells (Blann, 2000). The cytokine has been regarded a major mediator of inflammatory progression and tissue damage systematically and topically in sepsis. It has been demonstrated that overproduction of TNF-α in inflammation is pivotal to the induction of epithelial permeability (Ma et al., 2004) and endothelial barrier disruption (Marcos-Ramiro et al., 2014), along with tight junctional protein alterations of ZO-1, occludin, and claudin. It has been reported that increased production of TNF-α might lead to structural and functional alterations in pulmonary TJ function and acute lung inflammation in mice, whereas inhibition of TNF-α reduces the TJ permeability (Mazzon and Cuzzocrea, 2007) through promoting the expression of ZO-1 (Hermanns et al., 2010), occludin, and claudin-5 (Huang et al., 2016; Cerutti and Ridley, 2017), suggesting the important role of TNF-α on lung barrier dysfunction. In view of this, we speculate that UTI might play the protective role on TJs through reduction of TNF-α. To ascertain this, we pretreated the PMVECs with TNF-α receptor antagonist followed by LPS (An+LPS) and TNF-α treatment (An+TNF-α). Firstly, the present results have shown (**Figures 5D–F**) that the suppression or detrimental effects of LPS on production of tight junctional proteins were abrogated following administration of TNF-α antagonist into LPS stimulated PMVECs. This stands to reason that, among others, TNF-α is specifically involved in the process. Secondly, we have shown that PMVECs stimulated with TNF-α produced similar destruction effects on tight junctional proteins caused by LPS. More importantly, TNF-α receptor antagonist can increase the expression of tight junctional proteins (**Figure 6**) in TNF-α-treated PMVECs. Hence, it is justified to conclude that in the present CLP and PMVECs models, UTI acts to inhibit the expression of TNF-α in the endothelial cells, which would then arrest the degradation of junctional proteins and integrity of TJs. Consequently, it prevents the disruption of endothelia barrier and ultimately reduces the inflammatory edema of lung blood vessels following septic insult. The present results have shown that the effect of UTI on TJs was shared by TNF-α receptor antagonist, which suggested that UTI might exert its TJs-protective effect through anti-TNF-α by inhibiting the action of endogenic TNF-α and extrinsic TNF-α. While pulmonary endothelial cells have been regarded as a kind of inflammatory cells that produce TNF-α, massive pulmonary macrophages of M1 phenotype tend to accumulate in the septic lung tissues which may serve as the main source of TNF-α. In consideration of this, it is suggested that the decreased effects of TNF-α on TJs may be attributed to polarization of M1 macrophages into M2 phenotype with decreased production of TNF-α by UTI. Supporting this argument is the fact UTI has recently been shown to decrease the inflammatory response through polarization of macrophages from M1 into M2 phenotype (Liu et al., 2017). This, however, remains to be further investigated using our present experimental model.

To further clarify the underlying mechanisms, four TNF-α-associated signaling pathways, including inhibitor of NF-κB (IκB), p38 mitogen-activated protein kinases (p38 MAPKs), c-Jun N-terminal kinases (JNKs), and extracellular signal-regulated kinases (ERKs) in PMVECs subjected to different treatments were followed. IκB is the cytoplasmic inhibitory protein bounded to the transcription factor nuclear factor-kappa B (NF-κB) to keep it in an inactive state. The phosphorylation of IκB and its subsequent degradation allows the activation of NF-κB. p38 MAPK, JNK, and ERK all belong to a class of MAPKs. Both NF-κB and MAPKs are involved in directing cellular responses to diverse stimuli like proinflammatory cytokines and regulate cell functions of proliferation, gene expression, differentiation, cell survival, and apoptosis (Sabio and Davis, 2014). A common feature of the above mentioned signaling pathways is that they can be activated by proinflammatory cytokines like TNF-α. As for NF-κB pathway, it can further induce the inflammatory cascade and produce more proinflammatory cytokines (Menghini et al., 2016; Chiavaroli et al., 2017; Locatelli et al., 2018).

As depicted in **Figure 7**, compared with the control group, the phosphorylated form of IκB, p38, JNK, and ERK was increased significantly at 30 and 60 min following LPS treatment, whereas UTI treatment markedly depressed LPS-induced IκB, p38, JNK, and ERK phosphorylation. The present results are consistent with others as demonstrated in LPS-induced animal or human umbilical vein endothelial cells (Kim et al., 2009; Zhang et al., 2011; Li et al., 2016; Li X.F. et al., 2017). It is to be noted that these signaling proteins are also involved in the signal transmission of TNF-α as reported by others using different experimental paradigms (Sabio and Davis, 2014; Adam et al., 2016). It is therefore suggested that UTI achieves its protective effects on TJs through decreasing the phosphorylation of IκB, p38, JNK, and ERK induced by LPS. Furthermore, UTI can inhibit the production of endogenous TNF-α in PMVECs and use of the antagonist of TNF-α receptor has shown similar effects. Taken together, it is suggested that UTI ameliorates the permeability of pulmonary capillary endothelial cells challenged with sepsis through protection of TJ via inhibiting the TNF-α expression as well as NF-κB and MAPKs phosphorylation. However, the mechanism of the inhibition of NF-κB and MAPKs phosphorylation by UTI in sepsis-induced PMVECs remains to be further investigated.

## Conclusion

The present study has provided unequivocal experimental, morphological, and biochemical evidence that UTI is a potent agent that can attenuate the sepsis-induced permeability of pulmonary capillary endothelial cells through protecting the endothelia junctional proteins. The mechanisms responsible for these effects, at least in part, involve the inhibition of TNF-α expression via suppressing NF-κB and MAPKs pathways during sepsis.

## Author Contributions

H-DW and H-KZ conceived and designed the experimental project. MF, W-HZ, and W-LS carried out the *in vivo* (CLP) and *in vitro* (PMVECs and primary culture) experiments including immunofluorescence, Western blot analysis, measurement of lung injury score etc. Y-YD and D-MY assisted with the cell work. BX performed the CLP-model. MF and W-HZ prepared the first draft of the manuscript. All authors participated in discussion and editing, and approved the final manuscript.

## Conflict of Interest Statement

The authors declare that the research was conducted in the absence of any commercial or financial relationships that could be construed as a potential conflict of interest.
